# Unveiling the intersection: exploring obstetric violence in the Era of COVID-19 in Ecuador

**DOI:** 10.1186/s12889-023-17300-4

**Published:** 2023-12-21

**Authors:** Martha Fors, Paloma González, Santiago Jacho, Desirée Mena-Tudela, Kirsten Falcón

**Affiliations:** 1 One Health Group, Universidad de Las Américas, Avenida de los Granados, Vía a Nayón, Quito, Ecuador; 2 Universidad de Las Américas, Quito, Ecuador; 3 Departamento de Enfermería, Instituto Feminista. Universitat Jaume I, Castelló de la Plana, Spain

**Keywords:** Public health, Obstetric violence, Ecuador, COVID-19

## Abstract

**Introduction:**

Changes to healthcare delivery organization that have occurred to protect people from the virus COVID-19 may have led to harmful consequences to pregnant women intensifying obstetric violence. Prevalence of obstetric violence in Ecuador is high with a range between 30 and 70% approximately.

**Methods:**

This cross-sectional study was performed with the participation of 1298 women who answered EPREVO questionnaire from June 2021 to January 2022. Obstetrics characteristics’ relationship before and during COVID-19 were examined using Fisher exact test.

**Results:**

From 1598 respondents, 1284 (80.4%) gave birth before March 2020 Most of the participants (73.6%; CI:73.59–73.61) experienced obstetric violence during childbirth. Vaginal examination, enemas and genital shaving, episiotomy and cesarean section decreased significantly as well as rooming with the baby during the pandemic. Half of the women did not breastfeed the baby in the first hour but there were not statistically significant differences between giving birth before or during the infection from COVID-19.

**Conclusions:**

Levels of obstetric violence in Ecuador remains high but without major differences due to the COVID-19 pandemic, however some harmful medical practices considered as obstetric violence decreased but maybe to the fear to be infected by the virus.

## Introduction

Gender-based violence (GBV) is a universal problem. GBV is a complex social and public health problem, which represents a human rights violation [1]. In Ecuador, 56.9% of women have experienced psychological suffering, 35.4% physical and 32.7% sexual violence. In addition, 42.8% of women have experienced violence by their intimate partners [1].

Obstetric violence (OV) is a type of male violence that women suffer during their reproductive processes and it manifests through language and actions, undermining women’s autonomy [2, 3]. It entails interventions that dictate the sexual and/or reproductive process, often at the discretion of healthcare personnel. OV is the adjudication of a foreign body and the reproductive system of the woman, by a health professional who stipulates an asymmetric and insensitive relationship, at the same time sexist and degrading treatment is evidenced within health establishments, which give a decadent care of procedures offered [4, 5].

The World Health Organization has already stated that the treatment of women during pregnancy and especially during childbirth presents an disturbing picture [6]. As a result, some countries have legislated on this issue. There is a law in Ecuador that protects women to receive good quality gynecological and obstetric services, whether during their pregnancy, childbirth or postpartum period. It is considered unregulated, not following the correct protocols, denying her the right to continue with her reproductive life, implanting inadequate cultures or techniques, revealing professional secrecy, going overboard with medications, threatening life or autonomy, and mistreating physical aspects through invasive praxis. or psychological of the patient [7].

In Ecuador according to Arias, approximately one-third (32.8%) of the studied subjects had encountered obstetric violence [8]. Other studies in the country have shown high figures of obstetric violence in its different aspects [9, 10]. A national survey was conducted in 2019 reported 45.1% of obstetric violence in the country [11].

Available evidence indicates that the COVID-19 pandemic and response measures may lead to increased risk of gender-based violence (GBV) in general [12, 13]. In addition, some authors identified a possible increase in OV during the COVID-19 pandemic [14], although no evidence of this has been found.

Maternal health may be impacted by how the pandemic and its control measures influence the world economy, cultures, and healthcare infrastructure. The COVID-19 pandemic’s effects are anticipated to vary depending on a number of country-specific elements and be context-specific [15].

Individualization of the birth method is important, and cesarean sections should only be performed under medical supervision. Whether a woman or her child has a suspected, probable, or confirmed infection, they should be allowed to practice skin-to-skin contact, rooming in with their baby, and breastfeeding after giving birth [6].

Despite this, the number of women who suffer from OV is very large, of which they suffer various physical or psychological consequences depending on the patient. Among the most alluded to by themselves are impotence, episodes of panic attacks, low self-esteem, depression, loneliness, stress, anger, and anguish. Due to this treatment, the mothers lose the bond with their newborn, demonstrating repudiation or blaming him for the mistreatment he receives from the health personnel [16].

Increased ban of labor companionship, instant separation and seclusion from the newborn, needless operations carried out without a medical need (such as caesarean sections or labor induction), and the prevention of breastfeeding are examples of possible OV during COVID-19 [14].

Studies estimating the relationship between OV and COVID-19 pandemic are limited. There are no data about prevalence of OV during the three last years in Ecuador. Hence, the aim of the study is to investigate obstetric violence in women that gave birth before and during the pandemic in Ecuador.

## Methods

### Type of study and participants

This is cross-sectional study conducted from June 2021 to January 2022 during the COVID-19 outbreak. The inclusion criteria were as follows: women over 18 years of age who granted their informed consent to participate, have given birth in Ecuador from 1993 to nowadays, with internet access and were able to read and write in Spanish. The participant reported experience of only one childbirth. The exclusion criteria included incomplete questionnaire responses.

Women were invited to participate in the study through an online invitation using the Google Forms platform and posted on a popular digital newspaper called GK, which is the most widely read independent media source in Ecuador. This paper posted a link to the survey and the study was also promoting in Facebook.

### Data collection tool

Women completed EPREVO questionnaire. EPREVO was designed and validated by a group of experts from Universidad de Las Américas [17]. EPREVO has 30 items and it took 30 min to complete. The newspaper did not contact any of the participants, no personal or identifiable information was collected. EPREVO was based on 3 domains: Structural negligence: This factor measures the procedures carried out by health personnel who are inserted in an institutional structure that does not meet scientific evidence including physical violence, institutional and intentional oversights by health personnel (13 items). The second domain is Right to information measuring the rights of the women to have information about all the procedures that are carried out with her or her baby (9 items) a the third is Right to presence/Supportive care: This factor measures the woman’s right to be accompanied during labor, birth and postpartum. And the right of both (mother and newborn) to have immediate attachment after birth without complications (8 items) [17].

Socio-demographic data such as age, ethnicity, educational level or marital status were collected. To complete the data collection, other obstetric data were also collected regarding the number of previous children, type of previous births, prenatal control or attendance to childbirth preparation classes.

The variable set as the main outcome was the prevalence of obstetric violence during COVID-19 infection. Each item of the questionnaire was considered a variable to be compared in women that gave birth before March 2020 when the pandemic begun and after that date. The difference was reported by each of the domains of the questionnaire.

### Sample size

To determine the sample size for this study, a ratio estimation formula was employed, with parameters set at prevalence = 0.50, d (precision) = 3%, and α = 0.05. Expected prevalence was chosen taking into account the ignorance of the number of women suffering of obstetric violence during a pandemic. Initially, a sample size of 1067 was calculated. Subsequently, accounting for a potential 30% loss, the sample size was adjusted to 1524. The study was conducted using a non-probabilistic sampling approach, administered anonymously through the digital newspaper GK.

### Statistical analysis

A descriptive analysis was performed for all variables with frequency, percentage or mean, standard deviation, maximum and minimum according to the nature of the variable. A bivariate analysis was also performed using the Chi-square test using contingency tables. Obstetrics characteristics’ relationship before and during COVID-19 was examined using Fisher exact test. The characteristics of the sample and the items of the questionnaire were compared between those who had experienced childbirth before the pandemics versus those who had their babies during the COVID-. Data were processed using the Statistical Package for the Social Sciences (SPSS) v. 26 (IBM, Armonk, NK, United States of America). A statistical significance level of *p* < 0.05 was assumed.

## Results

It was received 1598 responses of participants. From them, 1284 (80.4%) gave birth before March 2020. The majority of women experienced obstetric violence, with a prevalence of 73.6% (1176 over 1598, CI 73.59–73.61) enduring all two or the three types of violence There were not statistical differences between those women that gave birth before and after COVID-19 regarding types of violence established by EPREVO. All women in the sample had been violated in their rights to have the presence of a relative or supportive care during childbirth (Table 1).

**Table 1:** Descriptive statistics of variables related to obstetric violence according to EPREVO questionnaire

	Women Gave Birth before COVID Pandemic		Women Gave Birth during COVID Pandemic		Total	*p* value
	No.	%	No.	%		
**Structural negligence**						
Yes	1261	98.2	308	98.1	1569	0.51
No	23	1.8	6	1.9	29	
**Information rights**						
Yes	955	74.4	235	74.8	1190	0.46
No	329	25.6	79	25.2	408	
**Right to presence/Supportive care**						
Yes	1284	100.0		314	1598	-----
No	0.0	0.0		0.0	0.0	

Regarding types of medical interventions, there were no significant differences in the frequency of Kristeller Maneuver (before COVID-19: Yes = 27.8%, n = 135; during COVID-19: Yes = 22.4%, n = 22; *p* = 0.22) and in membranes rupture (before COVID: Yes = 28.7%, n = 368; during COVID-19: Yes = 29.3%, n = 92; *p* = 0.14). Regarding breastfeeding, 50.6% from those who gave birth before the pandemic and 44.3% of those that had their babies during COVID-19 pandemic stated that they did not feed the baby in the first hour (*p* = 0.01). Vaginal examination, enemas and genital shaving decreased significantly during the pandemic in more than 10%, 20%, and 25%, respectively (Table 2).

**Table 2:** Variables related to structural negligence

	Women Gave Birth before COVID Pandemic		Women Gave Birth during COVID Pandemic		
	No.	%	No.	%	
**Vaginal examination less than 4 h**					
No	696	54.2	212	67.5	
Yes	539	42.0	92	29.3	0.00
I do not remember	49	3.8	10	3.2	
Total	1284	100.0	314	100.0	
**Tied to the bed or stretched by the hands or feet**					
No	1081	84.2	267	85.0	
Yes	182	14.2	42	13.4	0.00
I do not remember	21	1.6	5	1.6	
Total	1284	100.0	314	100.0	
**Breastfeeding during the first hour of life of the newborn**					
No	650	50.6	139	44.3	
Yes	650	35.4	139	43.3	
I do not remember	25	1.9	1	0.3	0.01
Not because of mother/newborn health problems	155	12.1	38	12.1	
Total	1284	100.0	314	100.0	
**Kristeller maneuver**					
No	321	66.2	73	74.5	
Yes	135	27.8	22	22.4	0.22
I do not remember	29	6.0	3	3.1	
Total	1284	100.0	314	100.0	
**Genital shaving**					
No	180	37.1	65	66.3	
Yes	196	40.4	7	7.1	0.00
I do not remember	26	5.4	0	0.0	
I was already rasured	83	17.1	26	26.5	
Total	485	100.0	98	100.0	
**Enema**					
No	858	66.8	275	87.6	
Yes	343	26.7	29	9.2	0.00
I do not remember	83	6.5	10	3.2	
Total	1284	100.0	314	100.0	
**Membranes rupture**					
No	750	58.4	194	61,8	
Yes	368	28.7	92	29.3	0.14
I do not know/I do not remember	166	12.9	28	8.9	
Total	1284	100.0	314	100.0	
**Episiotomy**					
No	125	25.8	46	46.9	
Yes	349	72.0	50	51.0	0.00
I do not know/I do not remember	11	2.3	2	2.0	
Total	485	100.0	98	100.0	

*Fisher test

Negative comments increased significantly (before COVID-19: Yes = 35.3%, n = 453; during COVID-19: Yes = 35.0%, n = 110; *p* = 0.03), and the possibility of rooming decreased (before COVID-19: No = 43.0%, n = 552; during COVID-19: No = 31.8%, n = 100; *p* = < 0.01). The increase in feelings of humiliation or guilt, discrimination, and not being separated from the baby did not reach statistical significance. It was observed that being accompanied during labor increased, but without statistical significance (Table 3).

**Table 3:** Variables related to Right to presence/Supportive care

	Women Gave Birth before COVID Pandemic		Women Gave Birth during COVID Pandemic		
	No.	%	No.	%	
**Humilliation/Guilt**					
No	937	73,0	239	76,1	
Yes	320	24.9	73	23.2	0.16
I do not know/I do not remember	27	2.1	2	0.6	
Total	1284	100.0	314	100.0	
**Negative commentaries**					
No	795	61,9	203	64,6	
Yes	453	35.3	110	35.0	0.03
I do not know/I do not remember	36	2.8	1	0.3	
Total	1284	100.0	314	100.0	
**Discrimination**					
No	1093	85.1	283	90.1	
Yes	152	11.8	26	8.3	0.06
I do not know/I do not remember	39	3.0	5	1.6	
**Skin to skin**					
I didn’t have my baby skin to skin	522	40.7	128	40.8	
Yes, less than 1 h	475	37.0	114	36.3	
Yes, 1 h or more	90	7.0	35	11.1	
No, due to health problems of the mother or newborn	180	14.0	35	11.1	0.08
I don’t know / I don’t remember	17	1.3	2	0.6	
Total	1284	100.0	314	100.0	
**Rooming**					
No	552	43,0	100	31,8	
Yes	565	44.0	172	54,8	0.00
I do not know/I do not remember	13	1,0	0	0,0	
No, because health problems of the mother or the newborn.	154	12.0	42	13.4	
Total	1284	100.0	314	100.0	
**Being separated from baby**					
No	584	45.5	153	48.7	
Yes	515	40.1	118	37.6	
I do not know/I do not remember	14	1.1	1	0.3	0.46
Yes, because health problems of the mother or the newborn.	171	13.3	42	13.4	
Total	1284	100.0	314	100.0	
**Company during the hospitalización**					
No	552	43.0	125	39.8	
Yes	667	51.9	177	56.4	0.31
Not all the time	65	5.1	12	3.8	
Total	1284	100.0	314	100.0	
**Breastfeeding**					
I did not receive support	435	33.9	97	30,9	
if it was enough	459	35.7	113	36.0	
It was not enough	368	28.7	100	31.8	0.60
I don’t know / I don’t remember	22	1.7	4	1.3	
Total	1284	100.0	314	100.0	

*Fisher test

Not having information about medication at hospital admission, information about pain relief, and health-related issues concerning the mother or the baby, as well as photos taken without consent or information, did not reach statistical significance. Timely information about the rupture of membranes, information about breastfeeding, and explanations regarding the baby being taken away increased during the pandemic (Table 4).

**Table 4:** Variables related to information rights

	Women Gave Birth before COVID Pandemic		Women Gave Birth during COVID Pandemic		*p* value
	No.	%	No.	%	
**Information about general medication at hospital admission**					
None	405	53.1	110	56.1	
Opportune information	295	38.7	75	38.3	0.45
I do not know/I do not remember	62	8.1	11	5.6	
Total	762	100.0	196	100.0	
**Information about medication for pain control**					
None	211	28.9	51	29.3	
Opportune information	473	64.7	114	65.5	0.82
I do not know/I do not remember	47	6.4	9	5.2	
Total	731	100.0	174	100.0	
**Information about membranes rupture**					
None	198	53.8	50	54.3	
Opportune information	144	39.1	42	45.7	0.02
I do not know/I do not remember	26	7.1	0	0.0	
Total	368	100.0	92	100.0	
**Informed consent for cesarean section**					
No	154	19.3	21	9.7	
Yes	604	75.6	187	86.6	0.00
I do not know/I do not remember	41	5.1	8	3.7	
Total	799	100.0	216	100.0	
**Information to feed the newborn with other milk than breast milk**					
No	518	66.1	120	60.3	
Yes	215	27.4	73	36.7	0.01
I do not know/I do not remember	51	6.5	6	3.0	
Total	784	100.0	199	100.0	
**Information about the health status of the mother and newborn**					
None	343	26.7	82	26.1	
Opportune information	880	68.5	225	71.7	0.12
I do not know/I do not remember	61	4.8	7	2.2	
Total	1284	100.0	315	100.0	
**Explanation of why the baby was taken away**					
None	238	34.7	39	24.4	
Opportune explanation	415	60.5	115	71.9	0.02
I do not know/I do not remember	33	4.8	6	3.8	
Total	686	100.0	160	100.0	
**Information to take photos or videos**					
No	22	39.3	15	45.5	
Yes	31	55.4	17	51.5	0.78
I do not know/I do not remember	3	5.4	1	3.0	
Total	56	100.0	33	100.0	

*Fisher test

During COVID-19 pandemic there was a significant decrease in cesarean sections. (Table 5)

**Table 5:** Variables related to obstetric history according period of childbirth

	Women Gave Birth before COVID Pandemic		Women Gave Birth during COVID Pandemic		
	No.	%	No.	%	
**Antenatal classes**					
No	866	67.4	214	68.2	0.84
Yes	418	32.6	100	31.8	
Total	1284	100.0	314	100.0	
**Antenatal care**					
No	52	4.0	6	1.9	
Yes	1232	96.0	308	98.1	0.09
Total	1284	100.0	314	100.0	
**Complications**					
No	700	54.5	183	58.3	
Yes	561	43,7	125	39,8	0.46
I do not know/I do not remember	23	1.8	6	1.9	
Total	1284	100.0	314	100.0	
**Healthcare facility**					
Public	1095	85.3	269	85.7	
Private	189	14.7	45	14.3	0.92
Total	1284	100.0	314	100.0	
**Type of delivery**					
Vaginal birth	799	62.2	216	68.8	
Cesarean section	485	37.8	98	31.2	0.03
Total	1284	100.0	314	100.0	

*Fisher test

## Discussion

The present study analyzed the level of obstetric violence in Ecuador before and during COVID-19 pandemic. To do so, an online survey was used to measure the level of Obstetric Violence (OV) referred by the participant women using EPREVO instrument [17]. Obstetric violence is a human rights violation and a serious public health issue that manifests as careless, reckless, omitting, discriminatory, and disrespectful behavior on the part of medical professionals, which is made acceptable by the power relations that normalize and minimize its occurrence [6].

The prevalence of obstetric violence in this study was of 73.6, much higher than the reported by other authors in the country before COVID-19 infection [8–11]. There are no scientific published data of this indicator during the pandemic. In Spain the prevalence of obstetric violence during the pandemic was much lower than in Ecuador with 26% [18].

Our participants reported having experienced all types of OV while receiving care during pregnancy, childbirth, or the postpartum period in both public and private healthcare settings. All of them reported rights violations concerning the presence of a relative or supportive care during hospitalization. There were not statistical differences between Structural negligence or Information rights in the two periods. It is important to note that all the women in our study had right to presence/supportive care violations.

The investigation into the prevalence of obstetric violence holds profound implications for public health initiatives and policies. By shedding light on the frequency and nature of mistreatment experienced by birthing individuals during pregnancy, childbirth, and postpartum care, this research serves as a crucial catalyst for reforming healthcare systems.

Regarding the relationship of obstetric violence and COVID-19, it is important to note that the pandemic has had significant impacts on healthcare systems globally. Many hospitals and healthcare providers have had to implement changes and protocols to adapt to the challenges posed by it, including in the field of obstetrics [19, 20]. These challenges may have indirectly affected maternity care and potentially contributed to an increase in obstetric violence in some cases [9]. Some possible factors that could contribute to this increase include the restriction in the number of support persons or visitors allowed during childbirth [21, 22]. This policy was implemented to reduce the risk of COVID-19 transmission but may have resulted in women feeling unsupported or experiencing mistreatment. The increased use of personal protective equipment such as masks and face shields, during childbirth may have hindered effective communication between healthcare providers and women. This lack of clear communication could contribute to feelings of mistreatment or violation [18].

The first important finding of this study was that more than 73.6% of the surveyed women assured having suffered all type of OV while being attended during pregnancy, birth or the puerperium both in public and private centers. All of them were violated in their rights to have a presence or supportive care during the hospitalization. This shocking result is a far cry from previous findings that one-third of women in Ecuador had experienced OV and two-fifths had experienced obstetric and gynaecological violence [8]. This finding would reinforce the thesis that the pandemic situation exacerbated obstetric violence [14]. Future studies are needed to further investigate this relationship, although World Health Organization (WHO) data show that women receive violent support worldwide. Thus, they encounter mistreatment, contempt, abuse, neglect and violation of human rights by health personnel, especially during labor and birth [16].

Due to the COVID-19 outbreak, there are limits and interventions being introduced in birth that were unnecessary, unsupported by science, disrespectful of human dignity, and out of proportion to the goal of stopping the virus’s spread. They consequently constitute obstetric violence and include pointless procedures carried out against medical care [8].

In this study, vaginal examination, enemas and genital shaving as well as episiotomy decreased significantly in those patients who delivered during the pandemic. Is our consideration this decrease may be due to the fear of professional staff to be infected by the virus and did not have close involving with these patients. Fear of healthcare workers being infected by COVID-19 has had a significant impact on the quality of medical care in maternal health such as shortage of the personnel involved in the assistance of pregnant women [23, 24]. The pandemic has instilled a sense of apprehension and anxiety among pregnant women who are understandably concerned about their own health and the well-being of their unborn babies [21, 22]. This fear has resulted in avoidance of seeking timely and necessary medical care, including prenatal visits and hospital admissions for labor and birth [25]. Reluctance to attend health facilities, coupled with overcrowded health systems and scarce resources, may have created difficulties in ensuring optimal obstetric care. Future studies should explore this aspect further [26].

The fear of contracting COVID-19 has also led to limitations in the availability of support persons during childbirth, resulting in decreased emotional and physical support for laboring women [22, 27]. Additionally, the implementation of infection control measures, such as personal protective equipment requirements and restrictions on visitors, has further affected the patient-provider relationship and the overall birthing experience [28]., These factors could support the fact that in our study all women we analyzed were These factors highlight the need for strategies to address the fear of infection in future potential pandemics and to ensure the provision of high quality, safe and compassionate care to pregnant women, promoting their physical and mental well-being during this critical period. The approach to fear, the relationship with healthcare workers, care and the barriers that personal protections confer should be investigated in order to provide more compassionate and empathetic care in future pandemics.

Obstetric violence could have physical consequences that ranging from an inadequate breastfeeding initiation and the incorrect use of antibiotic are example of the consequences of OV [29]. Other negative consequences are reported by different authors [30–32].

Also, episiotomy has been a standard practice in recent years, it is evident that it produces more problems than benefits at the time of birth, for example: it does not facilitate the expulsion of the newborn, it does not prevent vaginal tears, instead, it has been related to a higher risk of tearing due to this procedure [18, 19].

Physiological changes in the puerperium predispose to a higher incidence with respect to psychological consequences, such as postpartum depression, which represents a high risk of mortality, either for the mother or for the baby, because if a treatment or support may end in complications such as suicide or filicide. In addition to this, post-traumatic stress syndrome can also be seen in the postpartum period, generating a decrease in oxytocin levels and an increase in adrenaline concentrations, which prevents the bond between mother and child from being strengthened.

Isolation of the newborn can interfere with breastfeeding and its advantages, disrupt innate and specific immune defenses, disrupt newborn physiology, stress mothers, and interfere with the birth of maternal milk to the infant. It can also double the burden on the health system by requiring care for women and babies separately [33].

A study on the indirect effects of the pandemic estimated a reduction in antenatal care by at least 18%, and possibly up to 51.9%, and a similar reduction in postnatal care [34].

Many cases of obstetric violence have been seen during the pandemic; women who have given birth alone, women who have not had the opportunity to receive epidural analgesia, women with suspected COVID-19 who underwent cesarean section as indicated in the first protocols available, women with instrumented births because these same protocols indicated that “given that during active pushing the exhalation is greater and the effectiveness of the masks cannot be assured, we will try to shorten as much as possible the active phase of the expulsion (kiwi, forceps, etc.)”, women with suspected COVID-19 separated from their babies, disfavoring immediate skin-to-skin and without initiating breastfeeding. …)”, women with suspected COVID-19 separated from their babies, favoring immediate skin-to-skin contact and not initiating early breastfeeding [21, 35].

As previously mentioned, the recommendations aimed to continue guaranteeing rights even in the context of a pandemic. However, this study has shown that the rights most affected during the pandemic were the decline in antenatal care and access to birth monitoring. Moreover, we are reporting a decrease in the number of cesarean sections. Cesarean sections are linked to a decrease in exclusive breastfeeding and are risk factors for postnatal depression and post-traumatic stress disorder after childbirth [36]. Despite the decrease, the number of caesarean sections remains high and more needs to be done to improve these ratios.

COVID pandemic has generated changes in health systems and in our environment, care for women during childbirth was seen as something secondary and little attention was paid to it. At some point, couples were prevented from entering the birth rooms, so that in the harshest days of the pandemic, some women had to give birth alone in a hostile and threatening hospital environment [37].

Being a woman in times of pandemic, has also meant having more consequences at the level of mental and emotional health, they were more affected by major depression and anxiety problems, especially young women, and again suggests a possible relationship with the clear inequality, and the repercussion of social and economic consequences that affect women more [38]. These intersectionalities are deeply related to the increased risk of obstetric violence [21].

From the analysis carried out, the need to problematize the criteria that are taken into consideration when organizing and planning a medical care protocol for the situation of childbirth, birth and hospitalization is evident. This is mentioned because contradictions have been found between a usual care protocol and the COVID-19 health care protocol, and they make it visible that the reasons for some decisions made by health professionals are not based on the well-being of the patient and her environment but to personal and institutional interests such as economics and time control.

Public health strategies can focus on improving healthcare provider training to foster respectful and empathetic care, ensuring informed consent practices, and implementing robust reporting mechanisms to address instances of obstetric violence promptly. Addressing this issue not only enhances the quality of care for pregnant individuals but also promotes a healthier societal outlook on childbirth, fostering trust and confidence in healthcare systems. Ultimately, the practical implications of this research revolve around creating safer, more compassionate birthing experiences, thereby positively impacting public health outcomes for both mothers and infants.

## Conclusions

Obstetric violence, a distressing phenomenon, has brought attention to the importance of respectful and compassionate treatment during childbirth. In Ecuador, levels of obstetric violence are still very high. The COVID-19 pandemic, while posing challenges to healthcare systems, has also prompted a reevaluation of routine practices, leading to a decrease in some unnecessary medical procedures like excessive vaginal examinations, genital shaving, episiotomy and cesarean section. However, the possibility to have support of relatives or to stay with the newborn in the same room was limited. As we move forward, it is essential to prioritize respectful and evidence-based practices in obstetrics, ensuring the well-being and autonomy of women during this important life event.

### Limitations

The design of this study does not allow the establishment of cause-and-effect relationships, the observed associations are difficult to say if they are causal or simply reflective of correlations. This study also relies on self-reported data, which introduces the possibility of recall bias. Participants who agreed to take part may not be fully representative of the overall population of interest, it was limited to those with internet access and to the newspaper that promoted this research.

## Data Availability

The datasets used and/or analyzed during the current study available from the corresponding author on reasonable request.
